# Identification of differentially expressed small non-coding RNAs in the legume endosymbiont *Sinorhizobium meliloti* by comparative genomics

**DOI:** 10.1111/j.1365-2958.2007.05978.x

**Published:** 2007-10-26

**Authors:** Coral del Val, Elena Rivas, Omar Torres-Quesada, Nicolás Toro, José I Jiménez-Zurdo

**Affiliations:** 1 Department of Computer Science and Artificial Intelligence, E.T.S.I. Informatics, Universidad de Granada, Daniel Saucedo s/n18071 Granada, Spain; 2 Howard Hughes Medical Institute, Janelia Farm Research Campus19700 Helix Drive, Ashburn, VA 20147, USA; 3 Grupo de Ecología Genética de la Rizosfera, Estación Experimental del Zaidín, Consejo Superior de Investigaciones Científicas, CSICProfesor Albareda 1, 18008 Granada, Spain

## Abstract

Bacterial small non-coding RNAs (sRNAs) are being recognized as novel widespread regulators of gene expression in response to environmental signals. Here, we present the first search for sRNA-encoding genes in the nitrogen-fixing endosymbiont *Sinorhizobium meliloti*, performed by a genome-wide computational analysis of its intergenic regions. Comparative sequence data from eight related α-proteobacteria were obtained, and the interspecies pairwise alignments were scored with the programs eQRNA and RNAz as complementary predictive tools to identify conserved and stable secondary structures corresponding to putative non-coding RNAs. Northern experiments confirmed that eight of the predicted loci, selected among the original 32 candidates as most probable sRNA genes, expressed small transcripts. This result supports the combined use of eQRNA and RNAz as a robust strategy to identify novel sRNAs in bacteria. Furthermore, seven of the transcripts accumulated differentially in free-living and symbiotic conditions. Experimental mapping of the 5′-ends of the detected transcripts revealed that their encoding genes are organized in autonomous transcription units with recognizable promoter and, in most cases, termination signatures. These findings suggest novel regulatory functions for sRNAs related to the interactions of α-proteobacteria with their eukaryotic hosts.

## Introduction

Until a decade ago, only a handful of bacterial non-coding RNAs, besides tRNAs and rRNAs, had been identified as functional. Antisense RNAs transcribed from the opposite strand of their target mRNAs have long been studied as regulators of plasmids, phages or transposons functions (Wagner *et al*., 2002). Some other chromosomally located RNAs were identified by chance in the model bacterium *Escherichia coli* either as highly abundant transcripts that provide housekeeping functions to the cell (i.e. tmRNA, RNase P, 4.5S or 6S RNAs) or because of their interaction with specific proteins (i.e. CsrB RNA) (Wassarman *et al*., 1999). However, post-genomic research has revealed an unsuspected abundance and diversity of novel small-untranslated RNA molecules with key regulatory roles in both prokaryotic and eukaryotic organisms (Storz, 2002). In bacteria, a major class of these newly discovered small non-coding RNAs (sRNAs) regulates gene expression post-transcriptionally through baseparing with complementary sequence stretches, generally located in 5′-UTR regions of *trans*-encoded target mRNAs, thereby affecting the translation or stability of the message (Storz *et al*., 2004; 2005). Functional characterization of several known bacterial sRNAs has revealed that sRNA-mediated regulation of gene expression underlies the control of a variety of cellular processes, such as adaptation to abiotic stresses, quorum sensing or virulence (Wassarman, 2002; Lenz *et al*., 2004; Toledo-Arana *et al*., 2007). Remarkably, in Gram-negative bacteria the regulatory activity of most, if not all, of this group of antisense *trans*-acting sRNAs depends on their binding to the RNA chaperone, Hfq (Møller *et al*., 2002; Zhang *et al*., 2003).

To date, the majority of the prokaryotic sRNAs annotated in publicly available databases (i.e. Rfam database) have been identified in *E. coli* (Hershberg *et al*., 2003; Liu *et al*., 2005). Although some experimental strategies (i.e. shot-gun cloning of cDNAs from small-sized RNA transcripts or microarray analysis of total or Hfq-associated RNA populations) have contributed to the characterization of the *E. coli* RNome (Wassarman *et al*., 2001; Vogel *et al*., 2003; Zhang *et al*., 2003), most of the known bacterial sRNAs were first predicted by computational approaches and further verified experimentally by Northern analysis (Argaman *et al*., 2001; Rivas *et al*., 2001; Wassarman *et al*., 2001; Klein *et al*., 2002; Axmann *et al*., 2005; Livny *et al*., 2005; 2006; Pichon and Felden, 2005; Silvaggi *et al*., 2006; Mandin *et al*., 2007). The algorithms for the prediction of sRNA-encoding genes in bacterial genomes usually integrate several of the features common to the known sRNAs (Argaman *et al*., 2001; Carter *et al*., 2001; Rivas and Eddy, 2001; Rivas *et al*., 2001; Chen *et al*., 2002; Klein *et al*., 2002; Pichon and Felden, 2003; Livny *et al*., 2005): (i) location within the intergenic regions (IGRs); (ii) nucleotide sequence conservation among closely related species; (iii) presence of transcription and/or termination signals appropriately spaced over these regions; (iv) GC content bias; and (v) phylogenetic conservation of the putative secondary structure. QRNA and RNAz are two bioinformatic tools used to predict non-coding RNAs on the basis of comparative sequence data (Rivas and Eddy, 2001; Washietl *et al*., 2005). QRNA identifies base substitution patterns in pairwise alignments likely corresponding to a conserved RNA secondary structure rather than to a conserved coding frame or other genomic features (Rivas and Eddy, 2001), whereas RNAz combines an estimation of thermodynamic stability with structure conservation for the RNA predictions (Washietl *et al*., 2005).

Besides *E. coli*, computational genome-wide searches for sRNAs have been conducted for only a few of the sequenced bacterial species (Livny and Waldor, 2007). Thus, the vast majority of the bacterial RNomes remain to be characterized. *Sinorhizobium meliloti* is an agronomically relevant microorganism that establishes a nitrogen-fixing endosymbiosis with various forage legumes, including alfalfa (*Medicago sativa* L). In the proximity of the root hairs, the plant flavone luteolin specifically induces the synthesis and secretion of lipo-quitooligosaccharide signal molecules (Nod factors) in *S. meliloti* upon the transcriptional activation of the nodulation (*nod*) genes by the NodD1/NodD2 proteins (Gottfert, 1993; Patriarca *et al*., 2004). Subsequently, bacterial Nod factors trigger infection and organogenesis of new specialized organs in the plant, the so-called root nodules, where the microsymbiont differentiates into its nitrogen-fixing competent form, the bacteroid, within the plant cells (Patriarca *et al*., 2004 and references therein). At the time of publication of the *S. meliloti* genome, only the homologues of the tmRNA, 4.5S and RNase P RNAs were annotated as sRNAs either by the *S. meliloti* international consortium (Galibert *et al*., 2001) or in non-coding RNAs-dedicated databases (i.e. Rfam database). More recently, the *S. meliloti* tmRNA and the plasmid-borne *incA* locus which encodes an antisense sRNA that mediates incompatibility within the *repABC* family of α-proteobacterial plasmids have been experimentally characterized (MacLellan *et al*., 2005; Ulvé*et al*., 2007).

In this work, we have used two complementary strategies, eQRNA and RNAz, to search for novel sRNA-encoding genes in the IGRs of *S. meliloti*. Verification of eQRNA/RNAz predictions by Northern hybridization and RACE mapping led to the identification of eight previously unknown loci expressing small transcripts and organized in independent transcription units. Seven of the identified sRNAs are differentially regulated in free-living and symbiotic bacteria, which predicts novel regulatory functions for bacterial sRNAs in the α-proteobacteria–eukaryotes interactions.

## Results

### Prediction of sRNA-encoding genes in the IGRs of *S. meliloti*

**Fig. 1:**
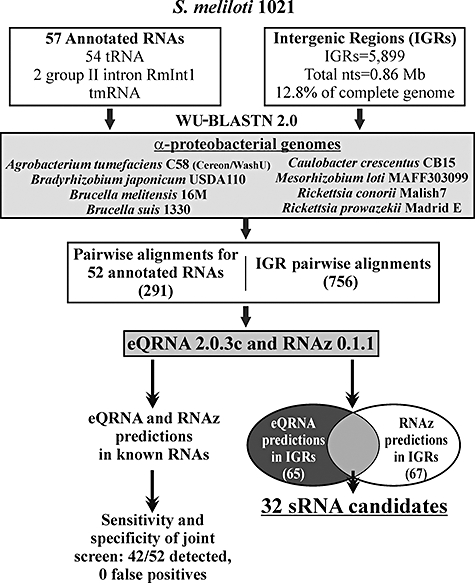
Strategy for the prediction of putative sRNA-encoding genes in *S. meliloti*. According to the existing *S. meliloti* annotation, two groups of alignments were generated by wu-blastn 2.0 comparisons against eight α-proteobacterial genomes using 57 known structural RNAs and the IGRs of *S. meliloti* as query sequences. Each alignment was scanned by eQRNA and RNAz. Sensitivity and specificity estimation of each program was assessed on known RNA alignments as described in the text. Overlapping eQRNA and RNAz predictions of structural sRNA loci from IGRs alignments were considered sRNA candidates. Eighteen putative untranslated *cis*-regulatory sequences mapping immediately upstream of annotated operons or ORFs were removed, leaving a total of 32 candidate sRNA loci.

The strategy used to search for putative sRNA-encoding genes in the IGRs of the *S. meliloti* genome is outlined in Fig. 1. The file with the IGRs contained 5899 sequences, totalling about 0.860 Mb, which represents 12.8% of the complete *S. meliloti* genome. The average IGR length was 146 nucleotides (nt), with 2792 nt being the longest. The IGRs with a length ≥ 50 nt and 57 known RNAs were used as queries to interrogate nine α-proteobacterial genomes (we used the two available annotations of the *Agrobacterium tumefaciens* genome) with wu-blastn. These comparisons generated two sets of pairwise alignments with 756 alignments for the IGRs and 291 alignments for the known RNAs, all with *E*-values ≤ 0.00001 and a length ≥ 50 nt (Table S1). These alignments were individually scanned by eQRNA and RNAz as described in *Experimental procedures*. By choice, predictions lacked strand specificity. In order to increase the sensitivity of the screen, candidates were selected when a signal was identify for either of the two strands. Overlapping eQRNA and RNAz predictions from the IGR alignments were collapsed into a single predicted RNA locus on the genome (Fig. 1). Conserved sequences immediately adjacent to the first 5′ nucleotide of annotated operons or open reading frames (ORFs), most likely corresponding to putative riboswitches or other *cis*-regulatory elements, were not considered further in this study, leaving a total of 32 candidate sRNA loci, which are listed in Table 1.

**Table 1:** Overlapping eQRNA and RNAz predictions of sRNA-encoding genes in *S. meliloti* 1021.

Candidate #^a^	Start	End	Predicted length	Flanking genes	Strand^b^	Observation^c^
**C7**	**201639**	**201834**	**196**	***polA/SMc02851***	**< >**	**Putative sRNA**
**C9**	**1398397**	**1398274**	**124**	***SMc01933/proS***	**< >**	**Putative sRNA**
C10	1411678	1411808	131	*celR2/rpmG*	> <	Matches to predicted ORFs
**C14**	**1667641**	**1667484**	**158**	***SMc02051/tig***	**< >**	**Putative sRNA**
**C15**	**1698744**	**1698610**	**135**	***SMc01226/SMc01225***	**< <**	**Putative sRNA**
**C16**	**1699021**	**1698812**	**210**	***SMc01226/SMc01225***	**< <**	**Putative sRNA**
C17	2098405	2098598	194	*SMc04270/SMc04273*	< <	Repeat Sm-2
C19	2357208	2356761	448	*SMc01857/SMc01856*	< <	RNase P RNA
C20	2398184	2398328	145	*SMc01608/ribH2*	> >	Matches to predicted ORFs
C21	2922966	2923066	101	*ctrA/SMc00653*	> <	Repeat Sm-4
**C22**	**2972265**	**2972118**	**148**	***SMc03975/SMc03976***	**> <**	**Putative sRNA**
C24	3074389	3074571	183	*pgm/glgX1*	> >	Repeat Sm-2
**C45**	**3105374**	**3105169**	**206**	***SMc02983/SMc02984***	**< >**	**Putative sRNA**

B29	24848	25053	206	*SMb20017/SMb20018*	< <	Repeat Sm-4
B30	56401	56555	155	*repC1/repB1*	< <	IncA
B31	66313	66506	194	*SMb20055/SMb20056*	> >	Repeat sequence
B32	73482	73615	134	*SMb20064/SMb20065*	< >	Repeat Sm-4
B33	231462	231581	120	*SMb20223/smc22–1*	> <	Repeat Sm-4
B34	334401	334598	198	*thuB/SMb20331*	> <	Repeat Sm-5
**B35**	**577732**	**577875**	**144**	***SMb20551/SMb20552***	**< >**	**Putative sRNA**
B36	783541	783640	100	*SMb21220/SMb21221*	< <	Repeat Sm-4
B37	908039	908163	125	*SMb21162/hutU*	> <	Repeat Sm-4
B38	1116680	1116860	181	*SMb21577/atcU2*	< >	Matches to predicted ORFs
B39	1230061	1230250	190	*SMb20993/SMb20994*	> <	Repeat Sm-4
B41	1249055	1249166	112	*xdhB2/SMb21676*	> <	Repeat sequence
B42	1373274	1373420	147	*SMb21444/SMb21445*	> <	Repeat Sm-4
B43	1525569	1525724	156	*SMb20720/SMb20721*	> <	Repeat sequence
B44	1669957	1670129	173	*paaX/SMb21642*	< <	Repeat sequence

A1	143338	143475	138	*SMa0255/SMa0257*	> <	Repeat Sm-5
A2	512130	512373	244	*SMa0922/traG*	> <	Matches to predicted ORFs
A4	1304125	1304268	144	*kdpA/SMa2335*	< <	Repeat Sm-5
A6	1328169	1328367	199	*SMa2355/SMa2357*	> >	Matches to predicted ORFs

a Letters before the number of each candidate denote genomic location: C, chromosome; B, symbiotic megaplasmid pSymB; A, symbiotic megaplasmid pSymA. Candidates experimentally tested are in boldface. The given candidate co-ordinates correspond to the RNAz prediction. Co-ordinates for the eight confirmed transcripts in bold indicate the gene orientation elucidated by Northern experiments. Orientation of the known RNase P and IncA RNAs is that reported in the Rfam database.

b Orientation of flanking genes. Genes present on the strand given in the *S. meliloti* 1021 genome database are indicated by (>) and those on the complementary strand by (<).

c Observations and additional information obtained from blast comparisons of the candidates. B38 had few short matches only to the *S. meliloti* genome.

The sensitivity and specificity of eQRNA and RNAz in the prediction of the 57 known *S. meliloti* RNAs were assessed as described in *Experimental procedures* (Table S2). In order to estimate false positives, we shuffled the alignments originated by the RNA genes while preserving the mutational and indel structure of the original alignments. Any shuffled alignment that scored as RNA was considered a false positive. Taking each method individually, the sensitivity on the known structural RNAs was 48/52 (92%) for eQRNA and 43/52 (83%) for RNAz (52 being the number of aligned RNAs; 5 tRNAs did not produce alignments with the required *E*-value). The number of false positives obtained with eQRNA was 4 out of the 52 aligned RNAs, and 8 out of 52 with RNAz. However, taking the overlapping predictions for both methods, we observed that while sensitivity was almost as good as for each of the individual methods, 42/52 (81%), specificity was substantially increased (no false positives for this test).

The sequences of the 32 intergenic joint sRNA candidates were reexamined to assess their conservation patterns and mapping to the *S. meliloti* genome (Table 1). A blastn comparison against all the available bacterial genomes using default parameters and the bioinformatic predictions as queries did identify the *S. meliloti* RNase P and IncA RNAs, used as positive controls, among the candidates. Thus, the remaining 30 candidates were initially regarded as novel putative *S. meliloti* sRNA loci. Seventeen of these 30 sequences, most of them located within the symbiotic megaplasmids, exhibited a large number of hits (usually > 30) to regions of the *S. meliloti* genome identified as repetitive non-genic elements (i.e. Sm or other repeats). Another five candidates were found to match or overlap ORFs recently predicted by Glimmer 2.0 in *S. meliloti* or its α-proteobacterial counterparts. The remaining eight sequences, seven of them located in the chromosome and one in the pSymB megaplasmid, had matches in unannotated regions of the *S. meliloti* genome and its α-proteobacteria blast partners covering most of or the full-length predicted sequence. They were considered the strongest candidates to encode true sRNAs and were further analysed by Northern hybridization.

### Experimental verification of the bioinformatic predictions

**Fig. 2:**
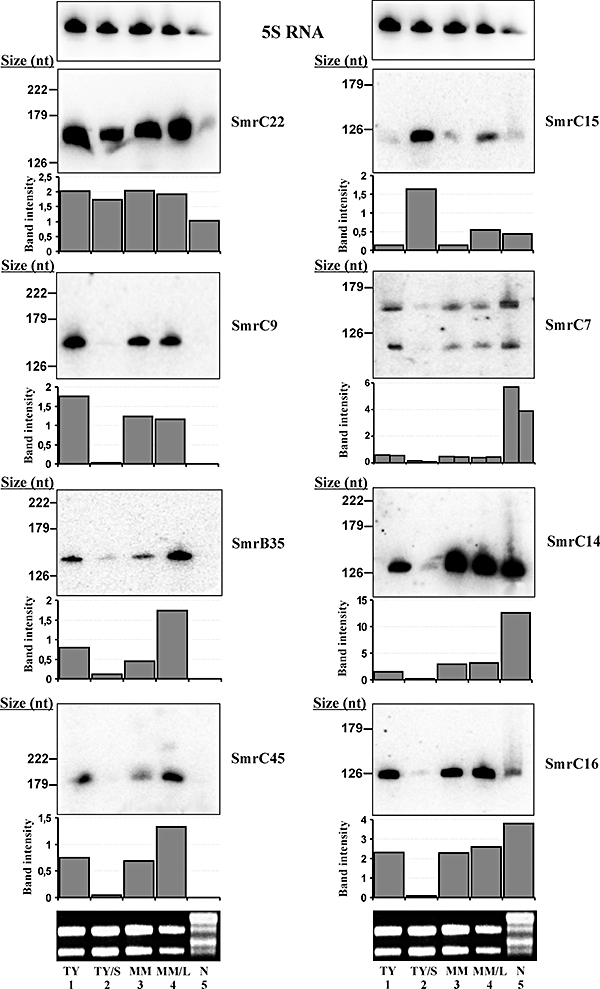
Northern analysis of the *S. meliloti* sRNAs. For each candidate, two strand-specific 25-mer oligonucleotides (Table S3) were used to probe RNA from free-living (1, log TY cultures; 2, stationary-phase TY cultures; 3, log MM cultures and 4, luteolin-induced log MM cultures) and endosymbiotic (5, nodules) bacteria. Exposure times were optimized for each panel; therefore, the signal intensity does not correlate with the relative abundance of each sRNA. Figure shows all the hybridization signals detected with each probe. The sizes (nt) of 5′-end-labelled pGEM DNA molecular weight markers (Promega Corporation), which were run in the gels with each set of samples, are shown on the left side of the panels. The range of transcript sizes resolved in each gel was approximately 600–25 nt. The complete set of Northern blots are provided in Fig. S1. Hybridization signals were quantified with the Quantity One software package, normalized to those of 5S RNA and plotted in a bar graph shown underneath each blot. Double bars in the graph for Smr7 correspond to the expression levels of the two RNA species detected for this transcript. Band intensities are expressed in arbitrary units.

For the eight selected eQRNA/RNAz-predicted loci (no strand specified), we designed 25-mer oligonucleotides (Table S3) for both strands to probe *S. meliloti* RNA obtained from log (TY and MM media), stationary (TY/S) and luteolin-induced (MML) cultures, as well as from *M. sativa* mature nodules (N). Luteolin was diluted in methanol and to rule out any effect of methanol in sRNA transcription, the MM was supplemented with this solvent at the same concentration as in luteolin-MM (0.1% v/v). All the hybridization signals were quantified with the Quantity One software package, normalized to those of the ribosomal 5S RNA in each biological condition and plotted in the bar graphs shown under the corresponding Northern blot (Fig. 2; the complete set of Northern blots for the two strands of all eight candidates are provided in Fig. S1). Hybridizations were repeated once for most of the candidates with the same or different oligonucleotide probe and similar results were obtained (data not shown). It should be noted that independent transcriptomic data showed an approximate 8-fold induction of 5S RNA expression in nodules when compared with free-living bacteria grown in TY medium (Barnett *et al*., 2004). Therefore, the expression levels of the *S. meliloti* sRNAs in nodule samples could even be underestimated in our assay.

Hybridization signals corresponding to small RNA transcripts (< 200 nt) were reliably detected for all the candidate sRNAs under study. Expression was detected with one of the strand-specific oligonucleotides but not with the complementary probe, which allowed us to assign the coding strand to all eight transcripts (Figs 2 and S1). The corresponding genomic locations were named *smr* for *S**.* *m**eliloti*RNA.

SmrC22 expression levels were only slightly different under all biological conditions tested and, therefore, it was considered as a constitutively expressed sRNA. All other candidates exhibited differential and specific expression profiles. Differential expression in logarithmic and stationary growth phases was found for these seven sRNA transcripts, with the majority being down- rather than upregulated during stationary phase. Accumulation in stationary phase was only found for the SmrC15 transcript.

All the sRNA genes were also expressed in bacteria grown in MM. However, luteolin moderately stimulated the expression of *smrB35*, *smrC45* and *smrC15* (∼4-, 2- and 3-fold respectively). Interestingly, expression of SmrC7 and SmrC14 increased ∼13- and ∼5-fold respectively in nodules when compared with free-living bacteria (log phase TY or MM cultures), suggesting the induction of these sRNAs during bacterial infection and/or bacteroid differentiation. Reliable hybridization signals in nodules were also detected for transcripts SmrC15 and SmrC16, whereas expression of *smrC9*, *smrB35* and *smrC45* genes was not detectable in endosymbiotic bacteria.

In summary, these experiments provided experimental validation for the bioinformatic screen and rendered seven *S. meliloti* sRNAs as putative riboregulators.

### Characterization of the transcription units of the *smr* genes

**Fig. 3:**
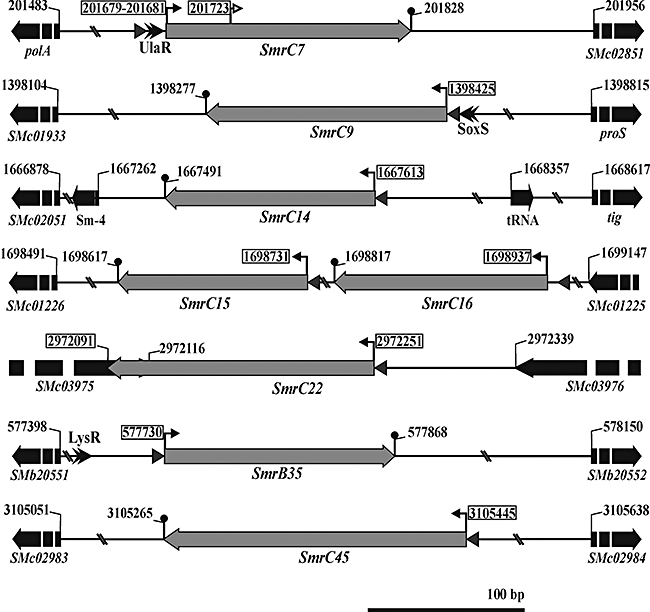
Genomic regions of the identified *S. meliloti* sRNA genes. The schematics (drawn to scale) summarize the bioinformatic predictions and the results of the experimental mapping. The *smr* genes are represented by grey arrows and the flanking ORFs by the dotted black arrows. Numbers indicate co-ordinates in the *S. meliloti* 1021 genome database. Experimentally determined 5′- and 3′-ends of the Smr transcripts are boxed. 3′-ends of the differentially expressed sRNAs were assigned to the last U in the consecutive stretch after extended stem-loops of Rho-independent terminators, which are denoted by black dots above the horizontal lines. The white arrowhead indicates the processing site for SmrC7. Putative σ^70^ promoters are indicated by single arrowheads, and putative transcription factors binding sites by double arrowheads.

The transcription units of the identified *S. meliloti* sRNA genes were further characterized by 5′-end mapping of the encoded transcripts and bioinformatic inspection of the promoter and termination regions. The results of these analyses are summarized in the diagram shown in Fig. 3. Details of the experimental mapping, the complete nucleotide sequences of the *smr* genes and the identified transcription signatures are provided in Figs S2 and S3.

The 5′-ends of the Smr transcripts were mapped by TAP-based RACE experiments to discriminate between primary 5′-ends and internal 5′ processing sites (Argaman *et al*., 2001). Major 5′-RACE products could be specifically obtained or increased after TAP treatment of the RNA samples, which enabled assignment of transcription start sites for the full-length transcripts detected in Northern experiments (Fig. S2). This procedure identified single 5′-ends for all the sRNAs except for SmrC7, which RACE analysis mapped the transcription initiation to two close G residues (Figs 3 and S3). From the six independent sequences obtained, five mapped to residue 201681 in the chromosome and one to the upstream residue 201679. Furthermore, an additional 5′-RACE product could be obtained from this transcript in both TAP- and mock-treated RNA samples (Fig. S2). This result suggests that the smaller RNA species detected in Northern experiments with the SmrC7 probe is a stable cleavage/processing product of the primary transcript. The sequence of this second RACE product mapped the processing site of SmrC7 to residue 201723 in the chromosome.

It is worthy to note that *smrC15* and *smrC16* genes are located in the same IGR and show a striking sequence similarity (84% identity) (Figs 3 and S3). However, specific probes for each sRNA detected transcripts of slightly different size with different expression profiles (Fig. 2). Consistent with *smrC15* and *smrC16* being organized in independent and differentially regulated transcription units, 5′-RACE experiments did identify transcription start sites for both sRNA genes.

Further bioinformatic analyses predicted DNA recognition sequences for the RNA polymerase sigma factor σ^70^ in the upstream regions of the *smr* genes compatible with transcription initiation at the mapped positions (Fig. S3). Furthermore, using matrices from known *E. coli* transcription factor binding sites with very restrictive parameters, we identified putative biding sequences for the transcription factors UlaR and SoxS in the promoter regions of the *smrC7* and *smrC9* genes respectively. Similarity searches and orthology analysis identified the genes *Smc02323* and *Smc00679* as the most probable *S. meliloti ulaR* and *soxS* orthologues respectively. In addition, 47 bp sequence stretches conforming to the consensus of the binding sites for the LysR-type transcriptional activator NodD (*nod* boxes) were specifically searched for in the promoter regions of the luteolin-induced *smrC15*, *smrB35* and *smrC45* genes. Although no conserved *nod* boxes were identified in these promoters, an inverted repeat structure built around the motif T-N11-A was found 55 nt upstream of the transcription start site of SmrB35 (Fig. S3). This characteristic sequence has been proposed as the specific binding site for the LysR-type proteins (Goethals *et al*., 1992).

Both the identified transcription start sites and the approximate size of the RNA species detected on gels enabled us to assign the termination of the seven differentially expressed transcripts to U-runs following hairpin loops of recognizable Rho-independent terminators (Fig. S3). Assuming that the 3′-end is located at the last U residue in the first consecutive stretch of Us, the sizes of these sRNAs are as follows: SmrC7, 148–150 nt (primary transcript) and 106 nt (processed transcript); SmrC9, 149 nt; SmrC14, 123 nt; SmrC15, 115 nt; SmrC16, 121 nt; SmrB35, 139 nt; and SmrC45, 181 nt.

The constitutively expressed *smrC22* gene lacked a recognizable termination signal at a distance from the mapped transcription initiation site consistent with the length of the detected sRNA and, therefore, 3′-RACE mapping was performed for this transcript. Sequences of the major amplification products placed the 3′-end at positions 2972091, 2972092 and 2972094 in the chromosome, thus overlapping the 3′ region of the flanking *SMc03975* gene transcribed in the opposite orientation (Figs 3 and S3). The length of the SmrC22 transcript as calculated from the most distant 5′ and 3′ end-points determined by RACE is 161 nt, in good agreement with the approximate size estimated from Northern blots.

### Conservation of the *S. meliloti smr* genes

The sequences of the eight *smr* genes were blasted using default parameters against all the 563 sequenced bacteria (http://www.ncbi.nlm.nih.gov; 20 July 2007) and used to interrogate the Rfam database version 6.1 (http://www.sanger.ac.uk/Software/Rfam) with the program Infernal (version 0.7) (Nawrocki and Eddy, 2007). The genomic regions of bacteria exhibiting significant homology to the query sequences were extracted for further analysis. The relevant results of these comparisons are summarized in Table S4, which reports the homologies obtained using blast. No additional homologies were found using Infernal and the database Rfam, thus revealing that none of the identified *S. meliloti* sRNAs showed significant similarity to known sRNAs from other bacteria. Indeed, as reported for other bacterial riboregulators, conservation of the newly identified *S. meliloti* sRNAs was found to be restricted to closely related bacteria. For comparison, using the *S. meliloti* sequence for RNase P RNA as query and the same search parameters, we identified the RNase P homologous genes in all proteobacterial groups and even in some Cyanobacteria.

The eight *S. meliloti smr* genes identified in this study were conserved, with identities ranging from 72% to 99%, in plant-associated bacteria, either symbionts (*S. medicae*, *Rhizobium leguminosarum* bv. viceae, *R. etli*, *Mesorhizobium* and *Bradyrhizobium* species) or pathogens (*A. tumefaciens*). *smrC22* and *smrC45* also shared similarity with IGRs in genomes of intracellular animal pathogens (*Brucella* species). *smrC22*, which exhibited a likely constitutive expression, was found to have significant sequence homology (*E*-values 6.0e−12–8.0e−54 and identities 72–94%) with genomic regions in more divergent α-proteobacteria (*Rhodopseudomonas palustris* and *Nitrobacter* species) besides the animal and plant pathogens and symbionts.

Overall, the genomic contexts of the *smr* genes were partially conserved. In many cases, wide conservation was limited to the sRNA-coding sequence and one flanking gene, whereas the promoter regions, the full-length IGRs and both flanking ORFs were only conserved in the more closely related bacteria. Translation of the *S. meliloti smr* genes and their α-proteobacterial homologues in all possible reading frames neither revealed significant coding potential (using a minimum ORF size of 30 amino acids with a methionine as the start codon), nor conservation of the putative short ORFs at the amino acid level. Both, their conservation pattern and translation features further support these loci as *bona fide* sRNA genes.

## Discussion

In this work, we have performed a whole-genome screen to identify novel sRNAs in the legume endosymbiont *S. meliloti* by a computational comparative genomics approach coupled to Northern experiments and RACE mapping. To our knowledge, this is the first report of such systematic search for structural RNAs in an α-proteobacterial representative.

### Bioinformatic prediction of putative sRNA-encoding genes in *S. meliloti*

We have used the unannotated portion of the *S. meliloti* genome and blast under stringent parameters (a smaller word size of 8 versus the default 11) to interrogate eight related α-proteobacterial genomes. Overlapping eQRNA and RNAz predictions were then taken in order to identify putative structural and conserved sRNAs. This approach has led to the identification of eight novel *S. meliloti* sRNA genes (*smr*), as confirmed by Northern experiments and RACE mapping.

It is worthy to note the reduced number of candidates predicted in our screen compared with most of the previous similar searches in bacteria (Carter *et al*., 2001; Rivas *et al*., 2001; Wassarman *et al*., 2001; Chen *et al*., 2002; Livny *et al*., 2005; 2006). This is the expected consequence of our searching strategy. First, we focused the screen on conserved sequences within IGRs larger than 50 nt and not adjacent to the 5′-ends of the annotated flanking ORFs. These criteria imply that putative sRNAs smaller than 50 nt, those unique to *S. meliloti*, *cis*-acting sRNAs transcribed at the same loci as the target gene (Wagner *et al*., 2002), and sRNAs processed from annotated mRNAs (Vogel *et al*., 2003) were not included or further analysed in the study. Second, our methodology takes into consideration the problem of false-positive predictions that affects the field of computational RNA gene finding by conducting a careful characterization of the (theoretical) reduction in the number of false positives that the combination of two methods (eQRNA and RNAz) would bring. Because the known *S. meliloti* sRNAs are highly dominated by tRNAs at present, the validation of the sensitivity of the screen was primarily carried out on this set of sRNAs. Although such a validation might not directly generalize for other sRNAs, it resulted in a screen with high rates of specificity and sensitivity. Alternative experimental approaches could add complementary information to the *S. meliloti* RNome. Indeed, *S. meliloti* transcriptome profiling using microarrays containing probes for the IGRs has revealed several hybridization signals in these regions that await interpretation (Barnett *et al*., 2004). Some could correspond to sRNAs that, for the reasons discussed above, escaped our screen. Despite the limitations, our experimental results support the combined used of eQRNA and RNAz as a reliable sRNA gene-finding strategy in bacteria with a small number of false-positive predictions.

### Most of the identified Smr transcripts exhibit potential riboregulator features

During its life cycle, *S. meliloti* alternates between a free-living phase in soil and rhizosphere and a differentiated endosymbiotic state within the host plant cells (Patriarca *et al*., 2004). These biological traits support the versatility of this bacterium to adapt to abiotic and symbiotic environments by regulatory mechanisms that are poorly understood. We have assessed the expression of the identified *smr* genes in cultured bacteria grown in different conditions as well as in mature symbiotic root nodules, mainly occupied by fully differentiated nitrogen-fixing bacteroids. Seven out of the eight candidates analysed exhibited differential expression profiles. These differentially expressed *smr* genes are organized in autonomous transcription units with predictable σ^70^ promoter and Rho-independent transcription terminator signatures. No additional DNA sequences recognized by alternative RNA polymerase sigma factors could be predicted in the promoter regions of these genes with reliable values. However, there are conserved sequence stretches in these upstream regions that remain unannotated and could contribute to the observed differential expression. Nonetheless, we did find sequences in the promoter regions of *smrC7*, *smrC9* and *smrB35* conforming to the consensus of the binding sites for the regulator for l-ascorbate dissimilation UlaR, the oxidative stress response regulator SoxS and the LysR-type of transcriptional regulators respectively (Goethals *et al*., 1992; Salgado *et al*., 2004). All together these findings predict a role for these seven Smr RNAs as *trans*-acting riboregulators in response to diverse abiotic and/or plant signals.

Similarly to what has been described for the known bacterial riboregulators, expression in a growth-dependent manner was also a common feature of these rhizobial sRNAs. However, whereas the majority of the characterized bacterial sRNAs accumulate at entry into stationary phase (Argaman *et al*., 2001; Wassarman *et al*., 2001), most of the Smr transcripts were preferentially expressed in exponentially growing bacteria. *S. meliloti* lacks a recognizable *rpoS* gene homologue, which in *E. coli* and other bacteria encodes the stationary phase/stress sigma factor σ^38^ (Repoila *et al*., 2003). Thus, our findings further support major differences in regulatory mechanisms underlying RpoS-dependent and RpoS-independent responses of bacteria to stress.

Although luteolin-regulated *nod* genes are well characterized in most of the rhizobial species, transcriptome analyses have identified some novel luteolin-induced genes which are also preferentially located in the symbiotic megaplasmids (Barnett *et al*., 2004). Accumulation of three of the *S. meliloti* small RNA transcripts identified in our study (SmrB35 and, to a lesser extent, SmrC15 and SmrC45) seemed to be moderately stimulated by this plant signal. The specific transcription signature found in the promoter region of the pSymB-borne *smrB35* locus suggests the regulation of this sRNA gene by a LysR protein. Nonetheless, whether the expression of this gene is specifically dependent on NodD activity, and the biological significance of the observed luteolin induction, remain open questions.

Proteome and transcriptome profiling have revealed a profound modification in *S. meliloti* gene expression during bacteroid differentiation (Djordjevic *et al*., 2003; Barnett *et al*., 2004; Becker *et al*., 2004). Our expression analysis revealed that two of the identified *S. meliloti* sRNAs are induced in endosymbiotic bacteria (SmrC7 and SmrC14). It is therefore tempting to speculate on the participation of these sRNAs in regulatory pathways leading to root infection and/or bacteroid differentiation.

Despite their differential biological traits, many α-proteobacteria share the capacity to establish a variety of long-term interactions with higher eukaryotes (Batut *et al*., 2004). Our results predict that the functional characterization of the *S. meliloti* sRNAs and their homologues will contribute to the unraveling of common strategies used by α-proteobacteria to infect and survive in eukaryotic cells.

## Experimental procedures

### Genomic sequences, extraction of IGRs and blast analysis

Sequences and annotations of the following α-proteobacterial genomes were downloaded from the NCBI (ftp://ftp.ncbi.nih.gov/genomes/Bacteria/, 9 March 2004): *S. meliloti* 1021, *Mesorhizobium loti* MAFF303099, *Bradyrhizobium japonicum* USDA110, *A. tumefaciens* C58 (versions Cereon and WashU), *Brucella melitensis* 16 M, *Brucella suis* 1330, *Caulobacter crescentus* CB15, *Rickettsia conorii* Malish7, and *Rickettsia prowazekii* Madrid E. The genomes of the legume symbionts *Rhizobium etli*, *R. leguminosarum* bv. viceae and *S. medicae* were not available at the time of initiation of the computer screen and, thus, they were not included in the initial analysis.

To generate a file containing the IGRs, the genes annotated in the *S. meliloti* database (http://bioinfo.genopole-toulouse.prd.fr/annotation/iANT/bacteria/rhime/) were subtracted from the whole genome sequence. Known genes were grouped in different categories: 6309 protein-coding genes (ORFs and insertion sequences), 66 RNAs [54 tRNAs, 3 copies of the rRNA operon (5S, 16S and 23S RNAs), tmRNA and 2 copies of the group II intron RmInt1 associated to the insertion sequence IS*Rm2011–2*], 374 repetitive elements (RIMES and Sm sequences), and a miscellanea of 267 sequences (motifs and other sequences). Three additional RNA genes coding for 4.5S (SRP), RNase P and IncA were not annotated at the time of the analysis and were kept in the IGR file as positive controls.

Alignments were made using IGRs from *S. meliloti* as queries in blast comparisons against the aforementioned eight α-proteobacterial genomes. We used wu-blast 2.0 (23 October 2003; http://blast.wustl.edu; López *et al*., 2003) with a word size of eight, and default parameters otherwise. Alignments with an *E*-value ≤ 0.00001 and a length ≥ 50 nt were kept and used as input data for eQRNA and RNAz. An additional set of alignments was obtained using 57 known RNAs (54 tRNAs, tmRNA and the 2 copies of RmInt1 associated to IS*Rm2011–2*) as queries. These RNA alignments were generated using similar blast parameters, and were used to assess the sensitivity and specificity of the computational analysis.

### eQRNA and RNAz analyses

QRNA analysis was performed using eQRNA version 2.0.3c. (ftp://selab.janelia.org/pub/software/qrna/). eQRNA uses three different probabilistic models to examine the pattern of mutations in a pairwise sequence alignment: RNA structure-constrained, coding-constrained (COD) and position-independent evolution (OTH). The alignment is then classified as RNA, coding or other, according to the Bayesian posterior probability of each model. The new eQRNA version applied in this study introduces an explicit probabilistic evolutionary model, which also incorporates insertions and deletions as previously described (Rivas, 2005). For each pairwise comparison, eQRNA selects the parameters of the model according to the degree of divergence observed in the alignment. We also used the program RNAz version 0.1.1 (http://www.tbi.univie.ac.at/~wash/RNAz) (Washietl *et al*., 2005).

The pairwise alignments were analysed by both eQRNA and RNAz using a window size of 150 nt and a window slide increment of 50 nt. Pairwise alignments were classified as RNA if they obtained a score of 3.5 bits or more. The RNA score represents the probability that the RNA model fits the data better than the OTH and the COD models combined. The RNA score is measured in bits and defined as the base-two logarithm of the ratio of the probability of the RNA model given the data to the sum of the probabilities of the COD and the OTH models.

eQRNA selected windows of these pairwise alignments with an RNA score of 3.5 bits or more. For RNAz, we selected windows with an RNA probability of 0.95 or more. The subset of windows (without strand specificity) predicted by both methods to have evolved under the constraint of a conserved RNA secondary structure was subjected to further analysis.

The above-mentioned cut-offs were selected by imposing to have at most 10 estimated false positives in the intergenic comparisons to the comparative genomes. We estimated the false-positive rate of the screen by randomly shuffling the pairwise alignments by windows (while maintaining the same substitution and indel patterns) and rescoring the shuffled alignments with the same programs. For eQRNA, we estimated the shape of the distribution of scores of random windows by generating a large number of shuffled windows from IGRs. The 10% tail of the distribution of scores of randomly shuffled intergenic alignment windows was fitted to a Gumble distribution (λ = 0.6123 and μ = −5.7247). A RNA log-odds posterior cut-off of 3.5 bits was inferred in order to obtain at most 10 false positives for the whole screen (which contains 2289 windows). For RNAz, a similar analysis revealed that the distribution of RNAz scores of randomly shuffled windows does not follow a well-characterized distribution. We determined empirically that an RNA probability cut-off of 0.95 roughly reproduced the target number of 10 estimated false positives in the whole screen.

The sensitivity for eQRNA and RNAz in *S. meliloti* was estimated by calculating the fraction of 57 known RNAs (54 tRNAs, tmRNA and 2 RmInt1) that were detected by both methods as RNAs using the above-determined cut-offs. The criterion for calling a predicted region RNA required that at least 50% of the length of the RNA is included in the prediction.

A compressed file (gzip.tar) with all the data corresponding to the computational screen can be downloaded from http://www.eez.csic.es/files/delVal.tar.gz.

### Prediction of transcription signatures

Sigma 70 (σ^70^) class I and class II promoters were gathered from the RegulonDB database (http://regulondb.ccg.unam.mx/) to build models of the RNA polymerase site using a neuro-fuzzy method (Bezdek, 1998; Cotik *et al*., 2005). The resulting models were then used to examine the upstream regions of the identified *S. meliloti* sRNA-encoding genes, with a false-discovery rate of < 0.001 (promoter search in http://regulondb.ccg.unam.mx/).

Putative transcription factors binding sites were searched for in the promoter regions using position weight matrices from RegulonDB, applying an extension of the Consensus/Patser program (http://regulondb.ccg.unam.mx/) and taking into account variations in the GC content of the IGRs of *S. meliloti* when compared with *E. coli* (Stormo, 2000; Salgado *et al*., 2004). Low thresholds corresponding to two standard deviations below the mean score obtained with the original *E. coli* model were used.

Rho-independent terminators were predicted with the TransTerm software (Kingsford *et al*., 2007; http://transterm.cbcb.umd.edu/index.php).

### Conservation analysis

wu-blastn 2.0 and the program Infernal version 0.7 (Nawrocki and Eddy, 2007) were used to search for homologues of the identified *S. meliloti* sRNAs in other bacteria and for similarities of these genes among the known structural RNAs deposited in the Rfam database respectively (http://www.sanger.ac.uk/Software/Rfam).

### Bacterial growth conditions and plant methods

Starter cultures of *S. meliloti* 1021 (OD_600_ 0.5–0.6) were diluted 1/100 in tryptone-yeast TY (Beringer, 1974) or defined MM (Robertsen *et al*., 1981) media, and cells were grown to log phase (OD_600_ 0.5) at 30°C. Bacteria in the stationary growth phase were obtained by incubation of TY cultures in the late exponential phase for a further 12–14 h. For flavonoid induction, bacteria were grown in MM broth to the log phase in the presence of 20 μM luteolin added from a 20 mM stock solution in methanol (Sigma Aldrich).

*Medicago sativa* L. ‘Aragón’ seeds were surface sterilized, germinated and planted in Leonard assemblies containing a nitrogen-free nutrient solution as described previously (Olivares *et al*., 1980; Fernández-López *et al*., 1998). Seedlings were inoculated with a *S. meliloti* 1021 bacterial suspension (∼10^6^ cells per seedling), and mature symbiotic root nodules were harvested 28 days after inoculation, immediately frozen in liquid nitrogen and stored at −80°C until RNA extraction.

### RNA isolation and Northern hybridization

Bacterial growth was stopped by adding 1/5 vols of stop solution (95% ethanol/5% phenol, v/v). Total RNA from cultured bacteria (20 ml broth) was then isolated by acid phenol/chloroform extraction and further treated with DNase I (Roche Diagnostics) as previously described (Cabanes *et al*., 2000). For endosymbiotic bacteria, ∼2 g of frozen nodules was ground and the powder suspended in 4 ml of NTES lysis solution (10 mM Tris-HCl pH 7.5, 100 mM NaCl, 1 mM EDTA, 1% SDS, 100 mM β-mercaptoethanol). RNA was further extracted with acid phenol/chloroform solution, ethanol precipitated and DNase I treated as described for cultured bacteria.

For Northern analysis, RNA samples (10 μg from free-living bacteria or 25 μg from nodules) were denatured for 5 min at 70°C in loading buffer (0.15% Bromophenol blue, 0.15% xylene cyanol and 48% formamide), subjected to electrophoresis on 6% polyacrylamide/7 M urea gels (16 × 16 cm) and transferred to nylon membranes by electroblotting. Labelled pGEM DNA molecular weight markers (Promega Corporation) were run in each gel to estimate the approximate sizes of the detected transcripts. Hybridizations were carried out at 42°C overnight using a 0.5 M phosphate buffer pH 7.2, 7% SDS, 10 mM EDTA, as hybridization solution and 50 pmol of specific 25-mer oligonucleotides, previously labelled at their 5′-ends by T4 polynucleotide kinase (New England Biolabs) with [γ−^32^P]-ATP (> 5000 Ci mmol^−1^; Amersham Pharmacia Biotech), as probes. The sequences of the oligonucleotides used as probes are provided in Table S3. After hybridization, membranes were washed by rinsing once with 2× SSC/0.1% SDS at room temperature, followed by three consecutive washes at 42°C for 15 min each with 2× SSC/0.1% SDS; 1× SSC/0.1% SDS and 0.1× SSC/0.1% SDS, and finally exposed to Phosphor Imager screens (Bio-Rad). Hybridization signal intensities were quantified with the Quantity One software package (Bio-Rad).

### 5′- and 3′-end mapping

5′- and 3′-RACE mapping were performed with some modifications as described by Argaman *et al*. (2001). Briefly, in 5′-RACE experiments, TAP treatment and 5′ RNA adapter ligation (5A, 5′-GCU GAU GGC GAU GAA CAC UGC GUU UGC UGG CUU UGA UGA AA-3′) were carried out with 15 μg of total RNA using the components of the FirstChoice RML-RACE kit (Ambion) and the reaction conditions specified by the supplier. Mock-treated RNA samples were also subjected to the adapter ligation and analysed in parallel. The ligated RNA (∼1 μg) was reverse-transcribed with the ThermoScript RT-PCR System (Invitrogen) using random hexamers as primers (10 min at 25°C, 60 min at 50°C and 5′ at 85°C), followed by RNase H treatment. The products of reverse transcription (1 μl of the RT reactions) were amplified with *Taq*-DNA polymerase by nested PCR with primer pairs (20 pmol of each primer) specific to the adapter (Ambion) and target sequences (Fig. S3). Cycling conditions were: 95°C 5 min; 35 cycles of 95°C 30 s, 60°C 30 s, 72°C 30 s and a final cycle of 72°C 5 min. 5′-RACE products were analysed in non-denaturing 10% polyacrilamide gels (Fig. S2) and cloned into the pGEM-T Easy vector (Promega Corporation). Bacterial colonies obtained after transformation were screened by colony PCR with T7 and SP6 primers, and 6–12 clones carrying inserts of the appropriate size were sequenced. For 3′-RACE mapping, 15 μg of RNA, previously dephosphorylated with calf intestine alkaline phosphatase (Ambion) was ligated as above to 100 pmol of a 3′-RNA adapter [3A, 5′-P-UUC ACU GUU CUU AGC GGC CGC AUG CUC-idT-3′ (Dharmacon Research); idT, 3′ inverted deoxythymidine]. Reverse transcription was carried out with the ThermoScript RT (Invitrogen) with 50 pmol of a primer complementary to the 3A RNA adapter (3RT, 5′-AGC ATG CGG CCG CTA AGA AC-3′) and the following reaction conditions: 20 min at 55°C, 20 min at 60°C, 20 min at 65°C and 5 min at 85°C with a final RNase H treatment. PCR amplification, cloning and sequence analysis of the 3′-RACE products were performed as detailed above.

## Supplementary material

This material is available as part of the online article from: http://www.blackwell-synergy.com/doi/abs/10.1111/j.1365-2958.2007.05978.x

(This link will take you to the article abstract).
