# Supplementary material for: Identification of differentially expressed small non-coding RNAs in the legume endosymbiont Sinorhizobium meliloti by comparative genomics
Source: Mol Microbiol. 2007 Oct 26;66(5):1080–91. doi: 10.1111/j.1365-2958.2007.05978.x (PMC2780559; doi:10.1111/j.1365-2958.2007.05978.x)
Supplement: Supplementary file 1 [file mmi0066-1080-SD1.pdf]

Table S1. Number of alignments found between *S. meliloti* and each BLAST partner after filtering.

Query[a]	Atc	Atw	Bj	Bm	Bs	Cc	Ml	Rc	Rp
**known RNAs**	43	43	29	43	43	25	43	12	10
**IGRs**	252	250	74	34	8	9	128	0	1

[a]Query sequences were: 57 known *S. meliloti* RNAs (54 tRNAs, 2 copies of group II intron RmInt1, and tmRNA) and Intergenic Regions (IGRs). Atc*, Agrobacterium tumefacien (Cereon);* Atw*, A. tumefaciens (WashU);* Bj, *Bradyrhizobium japonicum*; Bm, *Brucella melitensis*; Bs, *B. suis*; Cc, *Caulobacter crescentus*; Ml, *Mesorhizobium loti*; Rc, *Rickettsia conorii*; Rp, *R. prowazekii*.

Table S2. Sensitivity and specificity of eQRNA and RNAz independently and combined in detecting known *S. meliloti* RNAs.

	Blast Filter[a]	eQRNA[b]	RNAz[b]	eQRNA and RNAz[b]
**Real alignments**	52/57	48/52	43/52	42/52
**Shuffled alignments**	52/57	4/52	8/52	0/52

[a]BLAST comparisons detected 52 of the 57 known RNAs tested.
[b]A given RNA is considered detected if at least half of the length of the RNA is included in the prediction. In order to be able to estimate the specificity of both methods combined, both programs scored identical shuffled windows.

Table S3. Oligonuclotide probes used in Northern hybridizations

Candidate#	Sequence[a]	Target strand[b]
SmrC7	5'-ACCAGATGAGGACAAAGGCCTCATC-3'	<
	**5'-GATGAGGCCTTTGTCCTCATCTGGT-3'**	>
SmrC9	**5'-CGCGTGATCTTTAATCCGTTTCCGG-3'**	<
	5'-CCGGAAACGGATTAAAGATCACGCG-3'	>
SmrC14	**5'-TGCTTGATCTGATTGGCAACCGGGA-3'**	<
	5'-TCCCGGTTGCCAATCAGATCAAGCA-3'	>
SmrC15	**5'-GAGGAGAAAGCCGCTAGATGCACCA-3'**	<
	5'-TGGTGCATCTAGCGGCTTTCTCCTC-3'	>
SmrC16	**5'-ACTGGGAGGAGAAGCCACCAAAGAT-3'**	<
	5'-ATCTTTGGTGGCTTCTCCTCCCAGT-3'	>
SmrC22	**5'-TACTAGGTAGGTGGGCACCGTATGC-3'**	<
	5'-GCATACGGTGCCCACCTACCTAGTA-3'	>
SmrB35	5'-TGGTAAGCGATGATGAGGAAGGTCG-3'	<
	**5'-CGACCTTCCTCATCATCGCTTACCA-3'**	>
SmrC45	**5'-CCGCACCGTCGTTGCTTCAAGATGT-3'**	<
	5'-ACATCTTGAAGCAACGACGGTGCGG-3'	>

[a]Probes giving hybridization signals are in boldface.
[b]>, strand given in the *S. meliloti* 1021 genome database; <, complementary strand.

Table S4. Conservation of the identified *S. meliloti smr* genes in sequenced α-proteobacteria

sRNA gene#	BLAST partners[a]	E value	Identity (%)	Conserved stretch[b]	Sequence context conservation[c]
smrC7	*Smed* WSM	5.00e-57	93	1-150	2
	Rl bv. v. 3841	9.00e-16	77	26-149	2
	Re CFN42	2.00e-17	78	26-149	2
	At C58	2.00e-11	76	19-148	2
smrC9	*Smed* WSM	5.00e-63	97	1-149	2
	Rl bv. v. 3841	4.00e-39	88	149-1	2
	Re CFN 42	2.00e-36	87	149-1	2
	At C58	5.00e-32	84	149-1	2
	Ml MAFF303099	2.00e-12	78	38-149	2
smrC14	*Smed* WSM	8.00e-47	95	1-123	2
	Rl bv. v. 3841	9.00e-15	77	2-119	2
	Re CFN 42	7.00e-16	74	10-119	2
	At C58	4.00e-06	72	14-119	1
smrC15	*Smed* WSM	5.00e-49	98	115-1	2
	Re CFN42	6.00e-16	79	115-1	2
	Rl bv. v. 3841	2.00e-15	79	115-1	2
	At C58	3.00e-13	79	115-17	2
smrC16	*Smed* WSM	5.00e-49	97	1-121	2
	Re CFN42	2.00e-17	82	120-1	2
	Rl bv. v. 3841	2.00e-28	86	121-1	2
	At C58	7.00e-22	84	121-16	2
	Msp. BNC1	7.00e-08	74	4-102	2
smrC22	*Smed* WSM	1.00e-65	94	1-161	2
	At C58	2.00e-43	86	1-161	1
	Rl bv. v. 3841	3.00e-42	86	161-6	2
	Re CFN42	3.00e-41	85	161-6	2
	Msp. BNC1	4.00e-27	79	161-8	1
	Rp BisB5	1.00e-21	78	158-4	1
	Nw Nb-255	5.00e-20	80	158-37	1
	Ml MAFF303099	1.00e-21	77	161-1	1
	Nh X14	7.00e-18	81	148-37	1
	Bj USDA110	8.00e-17	81	37-143	1
	Bsp.ORS278	1.00e-13	80	143-37	1
	Oa ATCC49188	6.00e-19	76	161-8	1
	Bo ATCC25840	6.00e-19	76	13-161	1
	Bm 16M	6.00e-19	76	8-161	1
	Ba bv. 1 9-941	6.00e-19	76	161-8	1
	Bm bv. a. 2308	1.00e-13	75	127-1	1
	Bq Toulouse	6.00e-12	72	1-159	1
smrB35	*Re* CFN42	8.00e-35	87	1-139	1
	Oa ATCC49188	3.00e-33	84	139-1	1
	At C58	3.00e-22	80	139-9	1
smrC45	*Smed* WSM	3.00e-86	99	1-181	2
	At C58	2.00e-63	91	180-1	2
	Rl bv. v. 3841	4.00e-59	89	181-1	2
	Re CFN42	4.00e-59	89	181-1	1
	Msp. BNC1	4.00e-53	86	1-181	0
	Oa ATCC49188	2.00e-51	87	176-1	1
	Ml MAFF303099	2.00e-49	86	181-1	1
	Bm bv. a. 2308 (chr. II)	2.00e-50	87	1-176	1
	Ba bv. 1 9-941 (chr. II)	2.00e-50	87	1-176	1
	Bs 1330 (chr. II)	2.00e-50	87	1-176	1
	Bm 16M (chr. II)	2.00e-50	87	1-176	1

[a]***Smed***, *Sinorhizobium medicae*; ***At***, *Agrobacterium tumefaciens*; ***B*sp,** *Bradyrhizobium* sp; ***Bj***, *B. japonicum*; ***M*sp**, *Mesorhizobium sp.*; ***Ml***, *M. loti*; ***Re***, *Rhizobium etli*; ***Rl* bv. v**., *R. leguminosarum* bv. viceae; ***Ba* bv. 1**, *Brucella abortus* bv. 1; ***Bm***, *B. melitensis*; ***Bm* bv. a.**, *B. melitensis* bv. abortus; ***Bs***, *B. suis*; ***Bo***, *B. ovis*; ***Nh***, *Nitrobacter hamburgensis*; ***Nw***, *N. winogradskyi*; ***Rp***, *Rhodopseudomonas palustris*; ***Oa*,** *Ochrobactrum anthropi*; ***Bq***, *Bartonella quintana*. Unless otherwise indicated, matches are to the main chromosome of each BLAST partner.

[b]Numbers refer to nucleotide positions in the *S. meliloti smr* genes sequences which were used as queries.

[c]Conservation of IGR flanking genes (ORFs) in the α-proteobacterial partners. 0, no gene conserved; 1, one of the genes conserved; 2, both genes conserved.

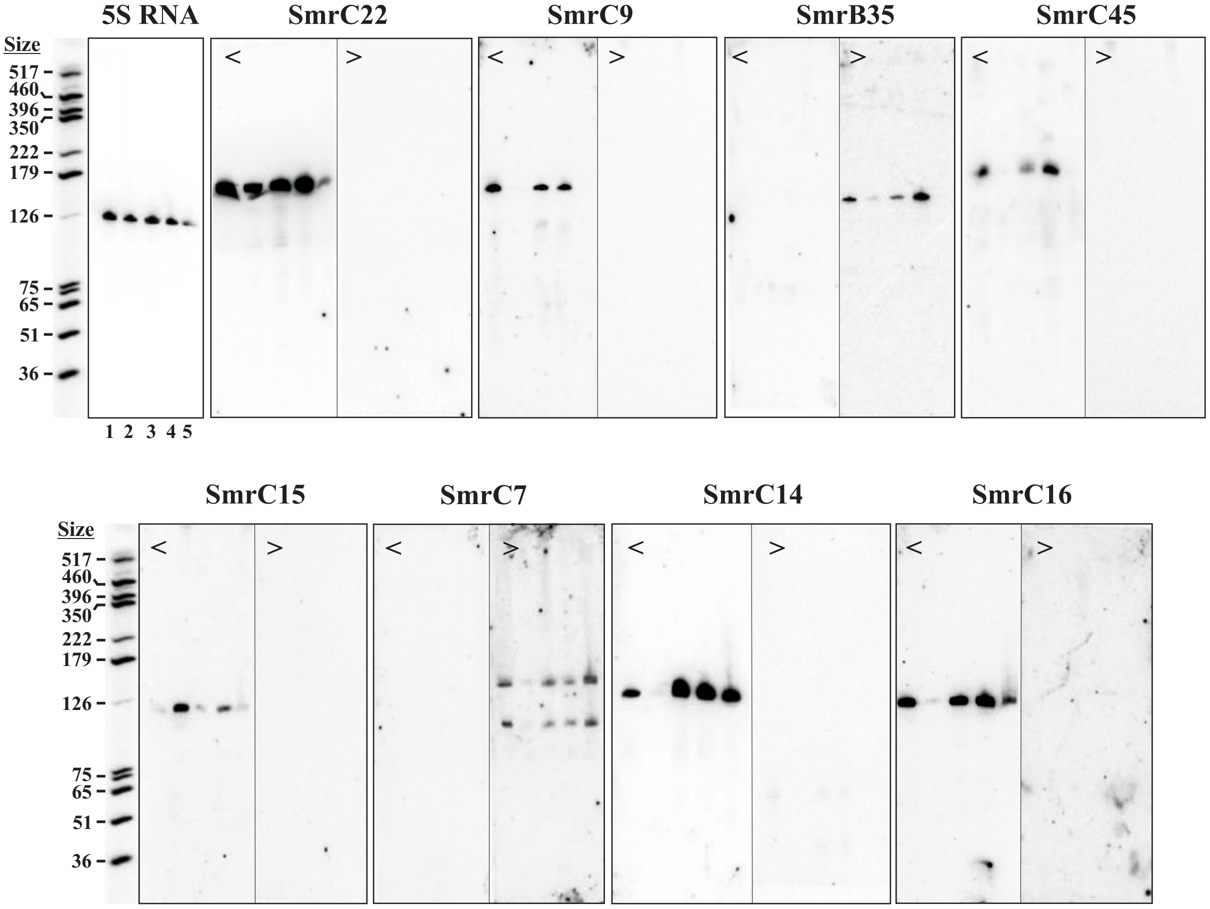

Fig. S1. Northern analysis of the *smr* genes. Membranes were probed with the oligonuclotides listed in table S3 to target both strands of the selected loci; >, strand given in the *S. meliloti* 1021 database, and <, complementary strand. Probe for the 5S RNA was 5'-TAAGACGAAGTACCATCGGCGCTGG-3'. Exposure times were optimized for each panel; therefore the signal intensity does not correlate with the relative abundance of each sRNA. pGEM DNA molecular weigh markers (5´-end labeled) were run in the same gel with each set of samples for approximate estimation of RNA transcript length. One lane of these markers is shown to the left of each panel. RNA samples were: 1, log TY cultures; 2, stationary phase TY cultures; 3, log MM cultures; 4, luteolin-induced log MM cultures, and 5, mature symbiotic root nodules (same order in all blots).

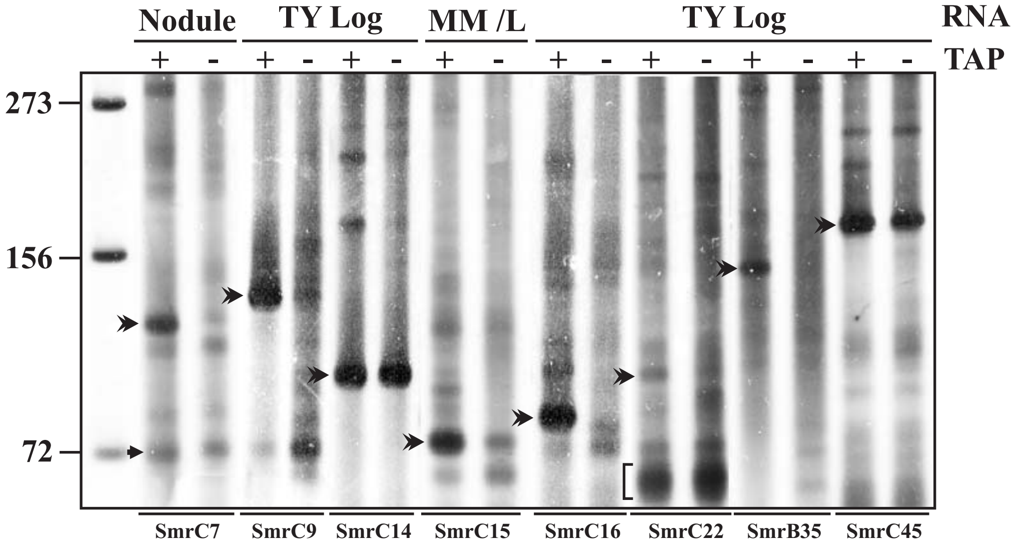

Fig. S2. 5'-RACE of the Smr transcripts. Silver stained polyacrilamide gel of the amplification products from TAP (+) and mock-treated (-) RNA samples. The source of RNA in each case is indicated on the top of the panel. Major RACE products specific or increased upon treatment with TAP are identified by a double arrowhead and represent transcription initiation sites. The single arrowhead indicates the processed SmrC7 transcript. The major PCR signal for SmrC14 from mock-treated RNA resulted from unspecific amplifications or PCR artefacts (none of the 10 clones analyzed contained a specific PCR product from this transcript) that were also generated from TAP-treated samples along with the expected specific RACE product (7 out of the 12 clones analyzed contained the specific RACE product for SmrC14). Major RACE products from SmrC22 (indicated by a bracket) in both TAP-treated and control samples identified heterogeneous 5'-ends most likely corresponding to degradation/processing intermediates (not detected in Northern gels). A minor RACE product specifically obtained from TAP-treated RNA identified the transcription start site for the full-length SmrC22 transcript detected in Northern experiments. This is likely the result of an inefficient ligation of the RNA adapter to this particular transcript.

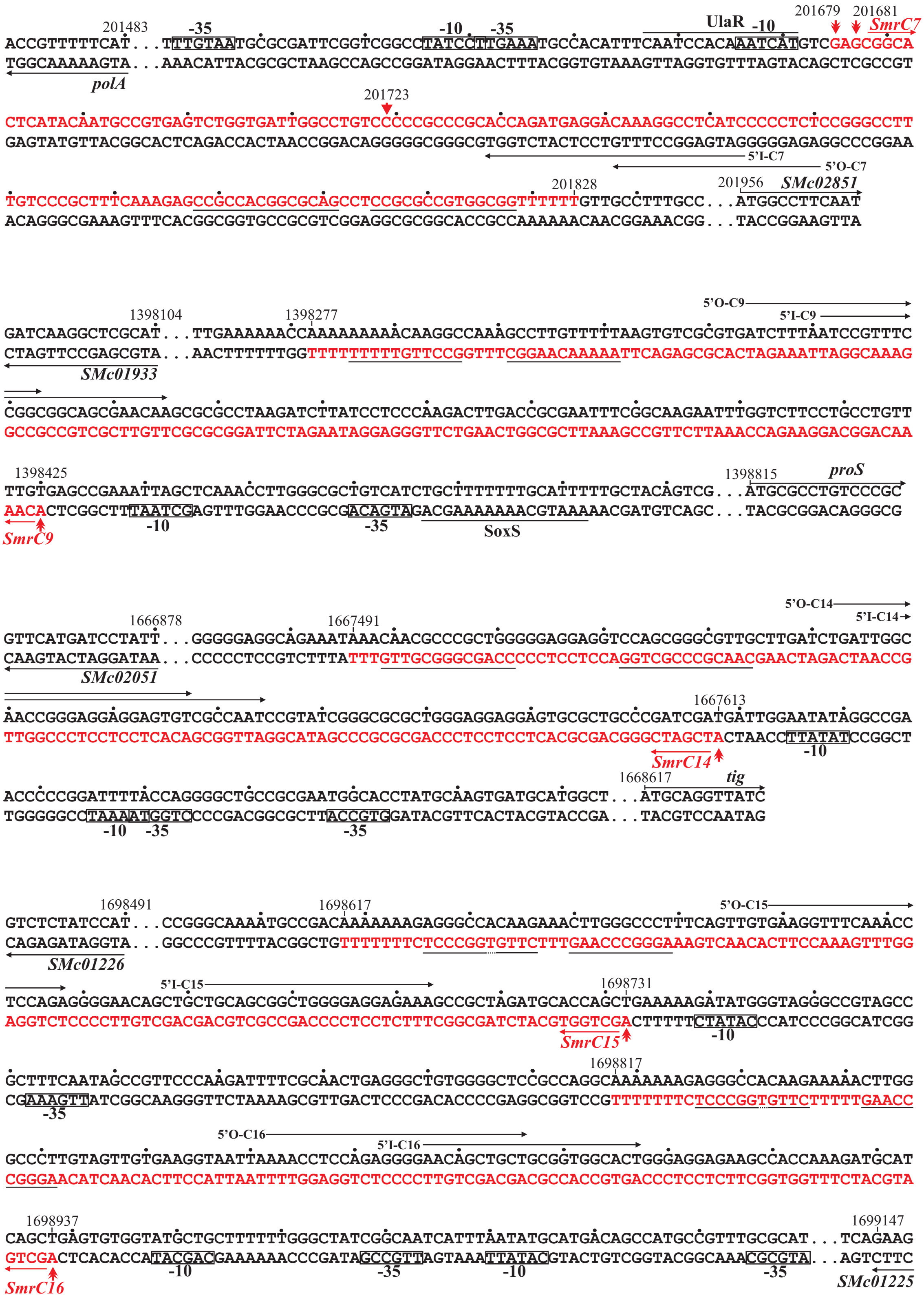

Fig. S3. Genomic regions of the *smr* genes. See legend in next page.

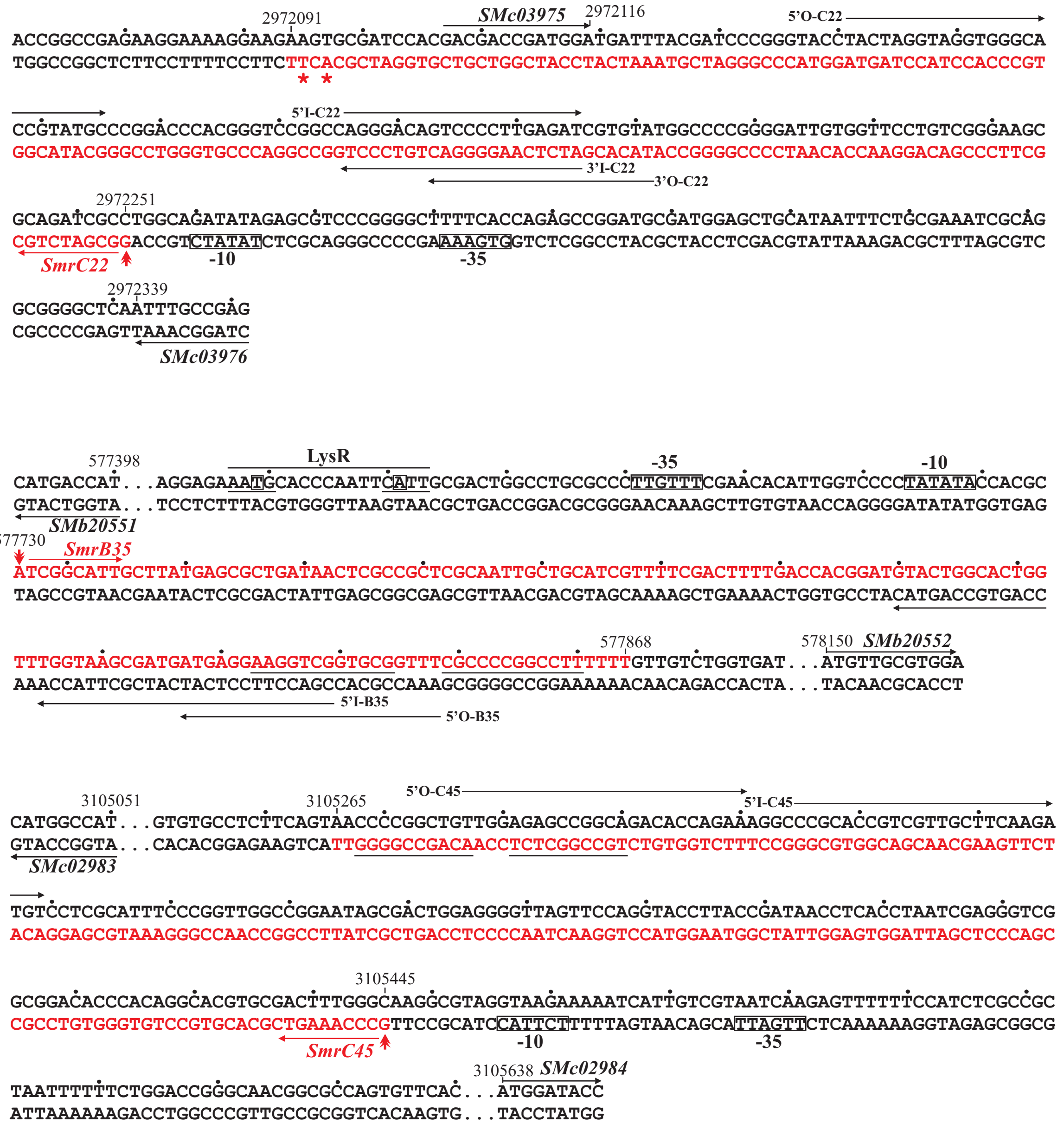

Fig. S3. Genomic regions of the *smr* genes. Nucleotide sequences of the *smr* genes are in red colour. The numbering denotes coordinates in the *S. meliloti* 1021 genome database. The ORFs flanking the *smr* genes are also indicated. The transcription initiation sites determined by 5'-RACE mapping are identified by a double arrowhead in red. The single arrowhead indicates the processing site of the SmrC7 transcript. The positions of the outer and inner primers (5'O/5'I) for the amplification of the 5'-RACE products by nested PCR are indicated by arrows. The 3'-ends correspond to the last uridine residue in the first consecutive stretch of Us after the stem-loop of the Rho-independent terminators. The inverted repeat sequences of each terminator are underlined. The 3'-end of the SmrC22 transcript, which lacks a recognizable termination signal, was determined by 3'-RACE mapping. Asterisks identify additional 3'-endpoints determined for this transcript. The positions of the outer and inner primers (3'O/3'I) for the generation of the 3'-RACE products are indicated by arrows. The -35 and -10 hexamers of all the predicted σ^{70} promoters are boxed. The putative binding sequences for the transcription factors UlaR, SoxS and LysR are indicated in the promoter regions of *smrC7*, *smrC9* and *smrB35* genes, respectively. Lines under the LysR recognition sequence identify the short inverted repeat sequence around the T-N11-A motif.
